# Nachhaltiges Alter(n) im Kontext des Klimawandels: Stand der Forschung und konzeptioneller Ausblick

**DOI:** 10.1007/s00391-024-02302-7

**Published:** 2024-04-25

**Authors:** Martina Brandt, Grit Höppner

**Affiliations:** 1 Sozialstruktur und Soziologie alternder Gesellschaften, Technische Universität Dortmund, Dortmund, Deutschland; 2 Fachbereich Sozialwesen, Katholische Hochschule Nordrhein-Westfalen, Piusallee 89, 48147 Münster, Deutschland

**Keywords:** Ältere Menschen, Klimakrise, Nachhaltigkeit, Teilhabe, Scoping Review, Older people, Environmental crisis, Sustainability, Participation, Scoping review

## Abstract

**Hintergrund:**

Die Alter(n)sforschung nimmt sich zunehmend der Herausforderungen im Zuge des Klimawandels an, behandelt jedoch eher die „Reaktionsbedarfe“ denn „Aktionsmöglichkeiten“ älterer Menschen.

**Material und Methoden:**

Auf Basis eines Scoping Review von 39 Fachbeiträgen werden Konstruktionen von Alter(n) im Kontext des Klimawandels und von Nachhaltigkeit analysiert und es wird auf existierende Verengungen hingewiesen. Aus diesen Überlegungen wird ein Modell entworfen, das erfolgreiches, aktives und nachhaltiges Alter(n) in Einklang bringen lässt.

**Ergebnisse:**

In der Fachliteratur werden ältere Menschen häufig als homogene, vulnerable Gruppe eingeordnet, die dem Klimawandel ausgeliefert ist. Im Kontext von Nachhaltigkeit kommt ihnen entweder die Rolle als zentrale Verursacher:innen des Klimawandels zu, oder sie werden als Teil der Lösung von Umweltkrisen verhandelt. Solche Verengungen werden in diesem Beitrag aufgelöst und es werden existierende Ambivalenzen in Lebenswelt und Forschung im Modell des „nachhaltigen Alter(n)s im Kontext das Klimawandels“ verbunden.

**Diskussion:**

Der Klimawandel kann nur gemeinsam bewältigt werden. Die Alter(n)sforschung kann auf Basis des vorgestellten Modells wichtige Beiträge zum Umgang mit Klimaveränderungen und zu (Re‑)Aktionen im Hinblick auf die Umweltkrise leisten.

## Abstract (en)

**Background:**

Research on age and ageing is starting to consider challenges related to climate change; however, most work focuses on reaction needs rather than action possibilities of older people.

**Material and methods:**

Based on a scoping review of 39 papers from the scientific literature the construction of age(ing) in the context of climate change and sustainability were analyzed and constrictions were revealed. Following these considerations, a model of “sustainable age(ing) in times of climate change” is proposed, which enables successful, active and sustainable ageing to be reconciled.

**Results:**

The scoping review shows that older people are often considered as a homogeneous, vulnerable group and more or less helpless in the face of climate change. In the context of sustainability, they are attributed the role of a central cause or as part of the solution for environmental crises. The focus is broadened and contradictions and ambivalences are reconciled in this model of sustainable age(ing).

**Discussion:**

Climate change can only be dealt with together. Research on age and ageing can support this on the basis of the model of sustainable age(ing) by providing important contributions to handling climate change and (re)actions regarding environmental crises.

## Hintergrund und Konzepte

Der Klimawandel ist eine der wichtigsten weltweiten Herausforderungen unserer Zeit. Mittlerweile ist eine Verhinderung klimatischer Veränderungen und damit verbundener bedrohlicher Umweltereignisse nicht mehr realistisch. Die Beschäftigung mit dem Umgang mit der Klimakrise gewinnt also rasant an Bedeutung [36] – auch in der Alter(n)sforschung. Dies zeigt der Anstieg an entsprechenden Fachbeiträgen in den letzten Jahren. Ein Scoping Review der Fachliteratur^1^ soll Aufschluss geben, wie das Verhältnis von Klimawandel, Nachhaltigkeit und Alter(n) in diesen Beiträgen dargestellt und welche Konstruktionen von Alter(n) dadurch vermittelt werden.

Auf Basis dieses Forschungsstandes wird ein Modell des nachhaltigen Alter(n)s entwickelt, das ungleiche Teilhabechancen in diesem Bereich in den Blick nimmt. Hier wird analog zur Nachhaltigkeitsforschung schwache und starke Nachhaltigkeit differenziert [17, 30].^2^ Unter schwacher Nachhaltigkeit wird ein Entwicklungsverständnis von Individuum und Gesellschaft gefasst, wonach der Verlust von natürlichen Ressourcen durch den Aufbau von technologischen Innovationen und Konsumentscheidungen aufgefangen und daher ökonomisch kompensiert werden kann. Demgegenüber wird mit starker Nachhaltigkeit auf Grenzen des natürlichen Wachstums, von Technik und Marktkräften hingewiesen und Verzicht, Suffizienz, Subsistenz und eine an den globalen Nachhaltigkeitsproblemen orientierte ökologische Lebensweise gefordert, um eine weltweit gerechte Inanspruchnahme von Ressourcen sicherzustellen [51]. Aus einer Teilhabeperspektive, im Sinne eines „Möglichkeitsraumes“ für Beteiligung [5], ist nachhaltiges Handeln abhängig von Ressourcen sowie persönlichen und gesellschaftlichen Bedingungen, die u. a. auch vom Alter abhängig sind oder gemacht werden [23]. Entsprechend zeigen Altersstudien (u. a. [41]) deutliche soziale und sozialräumliche Ungleichheiten in den Teilhabechancen Älterer, die sich auf erfolgreiches, aktives Altern [4, 37, 52] im Sinne nachhaltiger Beteiligung übertragen lassen. In unserem Scoping Review werden daher Merkmale verschränkter (vertikaler und horizontaler) sozialer Ungleichheiten (wie finanzielle und gesundheitliche Ressourcen, räumlich-zeitlicher Kontext, Geschlecht) berücksichtigt, die Einfluss auf Teilhabechancen von Älteren im Sinne nachhaltigen Handelns haben können [5].

Im Folgenden werden zunächst Material und Methode des Scoping Reviews vorgestellt. Das Material wird hinsichtlich des Verhältnisses von Klimawandel und Alter(n) und von Nachhaltigkeit und Alter(n) mit Blick auf ungleiche Teilhabe ausgewertet. Ergebnisse werden anschließend im Modell des „nachhaltigen Alter(n)s im Kontext des Klimawandels“ zusammengefasst, aus dem Forschungslücken und neue Perspektiven für die Alter(n)sforschung abgeleitet werden. Der Artikel leistet daher auch einen Beitrag zur konzeptionellen Entwicklung des Forschungsfeldes, die bisher erst punktuell erfolgte (siehe Leitbild des nachhaltigen Alter(n)s [51], Fundierung von Ergebnissen mittels geragogischer [7, 42], ökogerontologischer [49] praxistheoretischer [46] und menschenrechtsbasierter Ansätze [2]).

## Material und Methode

In einem Scoping Review der Fachliteratur der letzten 15 Jahren im deutsch- und angloamerikanischen Raum [45] wurden mittels der Schlagwörter Klima, Klimawandel, Naturkatastrophe, Nachhaltigkeit, ältere Menschen, Alter bzw. „climate“, „climate change“, „natural catastrophe/disaster“, „sustainability“, „older/elderly persons/people“, „age“ im Zeitraum 03.07.2023 bis 28.08.2023 über die Suchmaschinen PubMed, Scopus, GeroLit, Springer, Elsevier und Sage 46 Fachbeiträge identifiziert. Tab. 1 gibt einen Überblick zu den Charakteristika dieser Beiträge und zu den Kriterien, anhand derer 7 Beiträge nach einer Sichtung von der Analyse ausgeschlossen wurden. Die verbliebenden 39 Beiträge wurden hinsichtlich der oben genannten Fragestellungen ausgewertet. Wie in Tab. 1 ersichtlich, wurden die Beiträge Argumentationslinien zugeordnet (Reaktion auf oder Aktion aufgrund Klimawandel). Diese Zuordnung wurde im Auswertungsprozess weiter differenziert, zum einen nach der Art der vermittelten Alterskonstruktion (z. B. Vulnerabilität), zum anderen nach der Art der Darstellung von Älteren (Homogenisierung oder Differenzierung nach Gesundheit, Einkommen, Region, Geschlecht). Ergebnisse der Beiträge einer Zuordnung wurden mittels der inhaltlichen Synthese zur Beantwortung der Forschungsfragen zusammengeführt.

**Tab. 1:** Charakteristika der Beiträge

Quelle	Art der Publikation	Journal/Verlag	Design und Methode	Reaktion auf/Aktion aufgrund Klimawandel	Alterskonstruktionen	Homogenisierung/Differenzierung	Integration
Antal H, Bhutani S (2023) [1]	begutachteter Fachbeitrag	Ageing International	strukturierte Literaturrecherche	Reaktion	Vulnerabilität	Homogenisierung	ja
Ayalon L et al. (2021) [2]	begutachteter Fachbeitrag	American Journal of Geriatric Psychiatry	konzeptionell-theoretischer Beitrag	Reaktion	Vulnerabilität, Kritik an Ageismus	Differenzierung	ja
Ayalon L et al. (2023) The role of ageism in climate change worries and willingness to act. J Appl Gerontol 42:1305–1312							(a)
Ayalon L et al. (2023) [3]	begutachteter Fachbeitrag	Gerontologist	Scoping Review	Aktion	Kritik an Ageismus	Differenzierung	ja
Bateman H et al. (2015) Demography and climate change. Verlag der ÖAW, Wien							(b)
Birkmann J, Laranjeira K (2019) [6]	Fachbeitrag	ProAlter	quantitative Studie	Reaktion	Vulnerabilität, Resilienz	Differenzierung	ja
Bubolz-Lutz E et al. (2022) [7]	Sammelbandbeitrag	Kohlhammer	konzeptionell-theoretischer Beitrag	Aktion	Engagement, Wissen, Offenheit	Differenzierung	ja
Bundesministerium für Gesundheit (2023) [8]	Dokument der deutschen Bundesregierung	Eigenverlag	Präventionspapier/ Hitzeschutzplan	Reaktion	Vulnerabilität	Homogenisierung	ja
Carnes BA et al. (2014) [9]	begutachteter Fachbeitrag	Journal of Gerontology	strukturierte Literaturrecherche	Reaktion	Vulnerabilität	Homogenisierung	ja
Claßen T et al. (2013) [10]	Sammelbandbeitrag	Springer	strukturierte Literaturrecherche	Reaktion	Vulnerabilität, Resilienz	Homogenisierung	ja
Conrad K, Penger S (2019) [11]	Fachbeitrag	ProAlter	quantitative und qualitative Studie	Reaktion	Vulnerabilität, Resilienz	Homogenisierung	ja
Degen C et al. (2014) [12]	begutachteter Fachbeitrag	Zeitschrift für Gerontologie und Gereatrie (ZfGG)	quantitative Studie	Aktion	eingeschränktes Wissen, Besorgnis, umweltbewusst	Differenzierung	ja
European Commission (2023) [13]	Dokument der Europäischen Kommission	Eigenverlag	quantitative Studie	Aktion	eingeschränktes Wissen	Differenzierung	ja
Fernandez LS et al. (2002) Frail elderly as disaster victims: emergency management strategies. Prehosp Disaster Med 17:67–74							(c)
Filiberto D et al. (2010) [14]	begutachteter Fachbeitrag	Generations	quantitative Studie	Reaktion	Vulnerabilität	Homogenisierung	ja
Geck M (2023) [15]	Beitrag	ProAlter	Bericht	Aktion	Engagement, Wissen, Lebenserfahrung	Differenzierung	ja
Grewe HA et al. (2014) [16]	begutachteter Fachbeitrag	ZfGG	qualitative Studie	Reaktion	Vulnerabilität	Differenzierung	ja
Handmer et al. (2010) Review of fatalities in the February 7, 2009 bush fires. Centre for Risk and Community Safety. RMIT University, Melbourne							(a)
Haq G (2017) [18]	begutachteter Fachbeitrag	Public Policy & Aging Report	strukturierte Literaturrecherche	Reaktion	Vulnerabilität, Resilienz	Differenzierung	ja
Haq G, Gutman G (2014) [19]	begutachteter Fachbeitrag	ZfGG	strukturierte Literaturrecherche	Reaktion, Aktion	Vulnerabilität, Resilienz	Differenzierung	ja
Haq G et al. (2013) [20]	Projektbericht	Eigenverlag	quantitative Studie	Aktion	Wissen, Besorgnis, umweltbewusste Einstellung und Verhalten	Differenzierung	ja
Harper S (2019) [21]	begutachteter Fachbeitrag	Journal of Population Ageing	strukturierte Literaturrecherche	Reaktion	Vulnerabilität	Homogenisierung	ja
Herrmann A (2019) [22]	Fachbeitrag	ProAlter	strukturierte Literaturrecherche	Reaktion	Vulnerabilität	Homogenisierung	ja
Horton G et al. (2010) Drought, drying and climate change: emerging health issues for ageing Australians in rural areas. AJA 29:2–7							(a)
Leyva EWA et al. (2017) [25]	begutachteter Fachbeitrag	Journal of Nursing Scholarship	Scoping Review	Reaktion	Vulnerabilität, Forschungslücke Resilienz	Homogenisierung	ja
Lindemann U et al. (2018) [26]	begutachteter Fachbeitrag	ZfGG	quantitative Studie	Reaktion	Vulnerabilität, Resilienz	Homogenisierung	ja
Loughnan ME et al. (2014) Learning from our older people: pilot study findings on responding to heat. AJA 33:271–277							(a)
McCracken K, Phillips D R (2016) Climate change and the health of older people in Southeast Asia. Springer, Cham, S 29–52							(a)
McDermott-Levy R et al. (2019) [27]	begutachteter Fachbeitrag	Journal of Gerontological Nursing	strukturierte Literaturrecherche	Reaktion	Vulnerabilität	Homogenisierung	ja
Molinsky J, Forsyth A (2022) [28]	begutachteter Fachbeitrag	Housing Policy Debate	strukturierte Literaturrecherche	Reaktion	Vulnerabilität, Resilienz	Differenzierung	ja
Olfermann E et al. (2023) [29]	begutachteter Fachbeitrag	Pflegezeitschrift	strukturierte Literaturrecherche	Reaktion	Vulnerabilität	Homogenisierung	ja
Oven KJ et al. (2012) [31]	begutachteter Fachbeitrag	Applied Geography	strukturierte Literaturrecherche	Reaktion	Vulnerabilität	Differenzierung	ja
Pillemer K, Filiberto D (2017) [32]	begutachteter Fachbeitrag	Public Policy & Aging Report	konzeptioneller Beitrag, quantitative Studie	Reaktion, Aktion	Vulnerabilität, Umweltengagement als Lösung	Homogenisierung	ja
Pillemer K et al. (2010) [33]	begutachteter Fachbeitrag	Geronologist	quantitative Studie	Aktion	Umweltengagement als Lösung	Homogenisierung	ja
Pillemer K et al. (2011) [34]	begutachteter Fachbeitrag	Journal of Aging and Health	konzeptioneller Beitrag	Aktion	Umweltengagement als Lösung	Differenzierung	ja
Pillemer K et al. (2021) [35]	Fachbeitrag	Eigenverlag	Working Paper	Aktion	Umweltengagement als Lösung	Differenzierung	ja
Rückert-John J et al. (2012) [38]	Projektbericht	Eigenverlag	quantitative Studie	Aktion	nachhaltige Lebensführung als Lösung	Differenzierung	ja
Schade M (2020) [39]	Fachbeitrag	ProAlter	Bericht	Reaktion	Vulnerabilität	Homogenisierung	ja
Schlicht W (2020) [40]	Fachbeitrag	ProAlter	strukturierte Literaturrecherche	Reaktion	Vulnerabilität	Homogenisierung	ja
Steinfort-Diedenhofen J (2022) [42]	Sammelbandbeitrag	Kohlhammer	konzeptioneller Beitrag	Aktion	Umweltbildung als Lösung	Differenzierung	ja
Teti A et al. (2020) [44]	Fachbeitrag	ProAlter	strukturierte Literaturrecherche	Reaktion	Vulnerabilität	Differenzierung	ja
Wanka A (2020) [46]	Fachbeitrag	ProAlter	qualitative Studie	Reaktion	Vulnerabilität, Resilienz	Differenzierung	ja
Wanka A et al. (2014) [47]	begutachteter Fachbeitrag	ZfGG	quantitative Studie	Reaktion	Vulnerabilität, Resilienz	Differenzierung	ja
Wells N, Laquatra J (2010) [48]	begutachteter Fachbeitrag	Generations	strukturierte Literaturrecherche	Aktion	Green Housing als Lösung	Homogenisierung	ja
Welzer H, Kolland F (2014) [49]	Fachbeitrag	ZfGG	Einleitung zum Special Issue	Reaktion	Vulnerabilität, Resilienz	Differenzierung	ja
Wendt B et al. (2019) [51]	begutachteter Fachbeitrag	Soziologie und Nachhaltigkeit	theoretischer Beitrag	Aktion	Leitbilder der Alters- und Nachhaltigkeitsforschung	Differenzierung	ja

Ausschlusskriterien:

(a) Studie nicht im deutsch-/anglo-amerikanischen Raum durchgeführt

(b) Fokus der Studie liegt nicht auf Alter(n)

(c) Beitrag 2002 erschienen

## Ergebnisse

### Verhältnis von Klimawandel und Alter(n)

Unser Scoping Review verdeutlicht, dass in der Vermittlung des Verhältnisses von Klimawandel und Alter(n) der Fokus vor allem auf Konsequenzen von Extremwetterereignissen (insbesondere Hitze) für das Wohlbefinden, die körperliche und mentale Gesundheit sowie die Mortalität von älteren Menschen liegt [2, 9, 14, 21, 25, 27, 31, 32, 44]. Dabei sind Arbeiten zu finden, die Handlungsmöglichkeiten von Älteren im Hinblick auf die Folgen des Klimawandels thematisieren. Sie verdeutlichen, wie Ältere durch körperliche, emotionale und finanzielle Bewältigungsstrategien ihre Resilienz stärken und sich vor den Folgen von Extremwettern schützen können [11, 18, 19, 26, 28, 46, 47, 49]. Zudem werden präventive Maßnahmen (z. B. Baumaßnahmen) zum Schutz im urbanen Raum und in der stationären Pflege vorgestellt [1, 6, 8, 10, 16, 22, 29, 39, 40].

In diesen Debatten wird eher ein homogenes Altersbild vermittelt: Ältere werden als vulnerable Gruppe, die den Folgen des Klimawandels weitestgehend schutzlos ausgeliefert ist, konstruiert. Gleichwohl können Bewältigungsstrategien und strukturelle Maßnahmen sie unterstützen, um auf Folgen des Klimawandels zu reagieren. In weniger als der Hälfte der Beiträge wird beachtet, dass soziale Ungleichheiten im Hinblick auf Teilhabechancen, verknüpft mit Gesundheit, Einkommen, sozialräumlichen Gegebenheiten oder Geschlecht, Bewältigungsstrategien beeinflussen können [2, 6, 16, 18, 19, 28, 31, 44, 46, 47, 49]. Vorrangig werden Handlungsmöglichkeiten als Reaktionen auf den Klimawandel reduziert, Strategien im Sinne von Aktionen aufgrund des Klimawandels werden hingegen unter dem Stichwort Nachhaltigkeit thematisiert.

### Verhältnis von Nachhaltigkeit und Alter(n)

Das Verhältnis von Nachhaltigkeit und Alter(n) wird in der Fachliteratur an zwei Konstruktionen Älterer geknüpft, nämlich (1) als zentrale Verursacher:innen des Klimawandels aufgrund nichtnachhaltigen Handelns und (2) als Teil der Lösung von Umweltkrisen aufgrund von nachhaltigem Handeln. Die Vorstellung, *ältere Menschen seien zentrale Verursacher:innen des Klimawandels,* und der Vorwurf, sie hätten durch ihren Lebensstil in jüngeren Jahren maßgeblich zur heutigen Umweltkrise beigetragen, wird in der gerontologischen Literatur meist kritisiert [7]. Vereinzelt gibt es Arbeiten, die Schuld differenziert nach Gruppenzugehörigkeit zuschreiben. So seien Einstellungen zu Konsum und Freizeitgestaltung sowie der Lebensstil der Babyboomer-Generation oft mit hohen CO_2_-Emissionen verbunden, wenngleich gerade diese Gruppe verstärkt Wert auf Energieeffizienz im eigenen Wohnraum lege [19, 20]. Auch wenn CO_2_-Emissionen aufgrund eines veränderten Lebensstils im höheren Lebensalter abnähmen, würden hilfebedürftige Menschen in Altenpflegeeinrichtungen Ressourcen verbrauchen. Zudem wirke sich die häufige Tabletteneinnahme negativ auf die Wasserqualität aus [20]. Kognitiv beeinträchtigte ältere Menschen könnten die komplexen Prozesse des Klimawandels nur teilweise verstehen, und Sorgen aufgrund des Klimawandels setzten sich nicht unmittelbar in alltägliches Umweltverhalten um [12, 13]. Somit finden auch in der Fachliteratur altersdiskriminierende Vorstellungen ihren Ausdruck [3]. Zusammengenommen bezieht sich die Vorstellung, ältere Menschen seien zentrale Verursacher:innen des Klimawandels, auf das Verständnis von schwacher Nachhaltigkeit. Kollektive Problemlagen werden dabei individualisiert und Problemlösungen auf den einzelnen Menschen übertragen.

Die Vorstellung, *ältere Menschen seien Teil der Lösung von Umweltkrisen,* bezieht das Verständnis von schwacher und starker Nachhaltigkeit ein. Schwach nachhaltig würden Ältere agieren, wenn sie im Sinne von Nachhaltigkeitskompetenzen Energie und Wasser sparen, recyceln und weniger reisen [48, 51, 24]. Im Sinne von starker Nachhaltigkeit gewinnt die Vorstellung an Bedeutung, ältere Menschen seien Naturliebhaber:innen und Bewahrer:innen des Ökosystems, die durch zivilgesellschaftliches Engagement im Bereich des Umweltschutzes einen Beitrag leisten. In den Artikeln dazu wird betont, dass ein solchermaßen sinnstiftendes und gemeinsames Handeln sowie Umweltbildung soziale Integration fördern, Gemeinschaftsbildung unterstützen sowie Partizipation und Vernetzung mit Akteur:innen aus Zivilgesellschaft, Verwaltung und Politik ermöglichen würden [7, 15, 20, 32–35, 38, 42, 51]. Es gibt jedoch auch gruppenspezifische Kritik. So wird bemängelt, dass Ältere der unteren Bildungs- und Einkommensschichten sich seltener in Umweltorganisationen engagieren. Deshalb brauche es Untersuchungen zu den Barrieren, die sie an diesem Engagement hindern [34, 35]. Während Gesundheit, Bildung und Einkommen als Einflussfaktoren auf nachhaltiges Handeln genannt werden, wird der Einfluss von anderen Ungleichheitsdimensionen – etwa Geschlecht – nur selten erwähnt [51].

## Nachhaltigkeit und aktives Alter(n) – ein Widerspruch?

Die Verhältnisbestimmungen von Klimawandel, Nachhaltigkeit und Alter(n) erfolgen weitgehend über zwei Pole, nämlich Reaktion auf und Aktion aufgrund des Klimawandels. Diese Pole sind mit unterschiedlichen Alterskonstruktionen verbunden: Bei Reaktionen geht es hauptsächlich um die Vulnerabilität Älterer, im Hinblick auf Aktionen geht es um die Verursachung oder Lösung von Umweltkrisen durch Ältere. Diese Verengung nehmen wir zum Ausgangspunkt und erweitern den Blick auf die sozial ungleich verteilten Teilhabechancen von Älteren im Kontext des Klimawandels, indem zusätzlich Überlegungen zum schwach und stark nachhaltigen Handeln aus einer Prozessperspektive integriert werden.

Bringt man die Konstruktionen von Alter(n) im Kontext von Nachhaltigkeit mit der Lebenspraxis von Älteren in Verbindung, so ergeben sich zahlreiche Spannungsfelder. Schwache Nachhaltigkeit setzt einen autonom und selbstverantwortlich handelnden Menschen, der über finanzielle, körperliche und geistige Voraussetzungen verfügt, nachhaltige Konsumentscheidungen zu treffen, voraus [51]. Zum einen verfügen (auch) ältere Menschen nicht immer über diese Ressourcen, zudem dürfte vor allem die Generation der Babyboomer, die zur Zeit des so genannten Wirtschaftswunders aufgewachsen ist, den sorgsamen Umgang mit ökologischen Ressourcen nicht von klein auf erlernt haben. Zum anderen sprechen Leitbilder des erfolgreichen Alterns [4, 37] und aktiven Alterns [52] ältere Menschen zwar ebenfalls als eigenverantwortlich handelnde Subjekte an, die Umsetzung dieser Altersvorstellung in der Lebenspraxis erfolgt aber durch Investitionen in die eigene Kompetenz und Gesundheit, die oft nicht mit nachhaltigem Handeln vereinbar sind. Ältere orientieren sich an mancher Stelle dann *entweder* am erfolgreichen, aktiven Altern *oder* einem auf Nachhaltigkeit ausgerichteten Lebensstil. Ein Ideal, das erfolgreiches, aktives und nachhaltiges Alter(n) konsequent miteinander vereint, hat sich bisher weder in der alltäglichen Lebenspraxis von Älteren noch in der Alter(n)sforschung durchgesetzt [51].

Starke Nachhaltigkeit setzt ebenfalls einen selbstverantwortlich handelnden (älteren) Menschen voraus, wobei insbesondere gesundheitliche Ressourcen für eine Transformation hin zu einer nachhaltigen Lebensführung im Sinne von gesellschaftlich verantwortlicher Bürgerschaft wichtig sind [51]. Solche Ansprüche können (auch) ältere Menschen überfordern, zudem können ungleiche Lebensverhältnisse und regional unterschiedliche Teilhabemöglichkeiten eine nachhaltige Lebensführung erschweren. Zwar werden auf politischer Ebene strukturelle Lösungen anvisiert (z. B. Nachhaltigkeitsstrategie der Bundesregierung), das Umweltbewusstsein von Älteren für autofreie Stadt- und Wohngebieten hervorgehoben [38] und Zugänge zu nachhaltigen Lebens- und Wohnarrangements erleichtert (z. B. „healthy cities“, „age-friendly cities“, „green communities“ und intergenerationale Projekte [42, 48]). Allerdings werden ältere Menschen noch zu selten als Adressat:innen von politischen Nachhaltigkeitsprogrammen angesprochen [2] und erreicht [19].

## Modell des nachhaltigen Alter(n)s im Kontext des Klimawandels

**Abb. 1:**
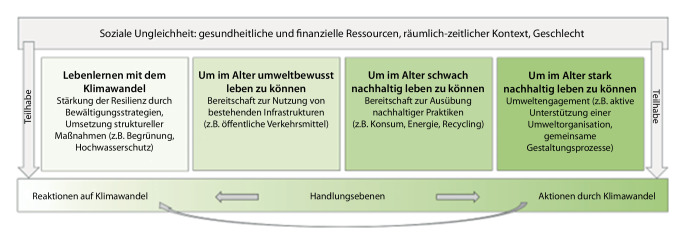
Modell des nachhaltigen Alter(n)s im Kontext des Klimawandels

Der bisherige Fokus der Fachliteratur auf Konsequenzen von Extremwettern für Ältere im Sinne von Reaktionen auf den Klimawandel greift entsprechend zu kurz. Werden hingegen Handlungen auch als Aktionen hinsichtlich des Klimawandels einbezogen, eröffnet dies neue Wege für Forschung und Teilhabe. Im Folgenden wird ein konzeptionelles Modell entwickelt, das die fruchtbare Verbindung zwischen Alter(n)s- und Nachhaltigkeitsforschung zum Ziel hat (Abb. 1).

Dieses Modell umfasst ein Spektrum an Handlungen, wobei der linke Pol Reaktionen auf und der rechte Pol Aktionen durch den Klimawandel markiert. Die von der Geragogik inspirierte Idee des Lebenlernens mit dem Klimawandel ist hier ebenso gemeint wie umweltbewusstes Handeln, das einen Beitrag zum Schutz des Klimas leistet. Schwach nachhaltig handeln Ältere, wenn sie punktuell nachhaltige Konsumentscheidungen treffen. Starke Nachhaltigkeit äußert sich in einem grundsätzlicheren Wandel, z. B. im Wirken über die eigene Lebensführung hinaus; dies kann Menschen zugutekommen, die (bisher ausschließlich) auf die Klimakrise reagieren (Abb. 1*Pfeil*).

Dieses Modell zeigt, dass Alterskonstruktionen im Kontext des Klimawandels vielfältiger sind und werden müssen, als dies die Fachliteratur mit dem Fokus auf Konsequenzen für die Gesundheit nahelegt. Ältere sind nicht nur vulnerabel und dem Klimawandel weitestgehend schutzlos ausgeliefert, sondern sie verfügen über die Fähigkeit zur Anpassung an den Klimawandel und können umweltbewusst und unterschiedlich stark nachhaltig eingestellt sein und handeln [15].

Das Scoping Review verdeutlicht, dass in der Verhältnisbestimmung von Klimawandel und Alter(n) sozial ungleiche Teilhabechancen nicht immer beachtet werden. In unserem Modell wird hingegen verdeutlicht, dass Ältere in vielfältigen Verhältnissen leben und ihnen unterschiedliche Teilhabemöglichkeiten zur Verfügung stehen; der Gesundheitszustand, das Einkommen, die zur Verfügung stehende Zeit und sozialräumliche Gegebenheiten können ein nachhaltiges Alter(n) beeinflussen. Studien aus der Nachhaltigkeitsforschung zeigen, dass Frauen umweltbewusster eingestellt sind als Männer und sich diese Einstellung teils im Umweltverhalten niederschlägt [50]. Das Scoping Review zeigt jedoch, dass Ältere im Kontext von Nachhaltigkeit meist geschlechtslos konstruiert werden – was erstaunt, sind es doch insbesondere ältere Frauen, die sich sichtbar in Klimabewegungen engagieren („Omas for Future“, „KlimaSeniorinnen“). In unserem Modell wurde daher Geschlecht als weiterer Faktor für nachhaltiges Alter(n) ergänzt.

Der Fokus auf soziale Ungleichheit und Teilhabe ermöglicht es, ein nachhaltiges Alter(n)sideal zu etablieren, das erfolgreiches, aktives Alter(n) konsequenter als bisher mit einer nachhaltigen Lebensführung verbindet. Zudem können Reaktionen auf den Klimawandel über eine bloße Teilnahme an infrastrukturellen Maßnahmen hinausgehen, wenn sie eine aktive Einbindung Älterer bewirken – so können soziale Teilhabe und nachhaltiges Handeln Älterer Hand in Hand gehen. Gleichwohl umfasst ein nachhaltiges Alter(n) auch biografische und generationenübergreifende Entwicklungen. Es stellt somit keinen ausschließlich altersbezogenen Handlungsauftrag dar, sondern eine kollektive Aufgabe. Der Klimawandel lässt sich nicht mehr verhindern, aber doch immerhin, so lässt sich hoffen, generationenübergreifend gemeinsam bearbeiten und gestalten.

## Schlussfolgerungen

Der Beitrag verdeutlicht die Notwendigkeit, Forschung zum Klimawandel nicht auf dessen Konsequenzen für Ältere zu reduzieren. Stattdessen ist es notwendig, Klimawandel, Nachhaltigkeit und Alter(n) zusammenzudenken, um Altersvorstellungen über und Handlungsmöglichkeiten von Älteren im Kontext des Klimawandels zu erweitern. Das vorgestellte Modell, in dem Ergebnisse eines Scoping Review zusammengefasst und weitergeführt werden, ist ein konzeptioneller Schritt in diese Richtung. Das Scoping Review zeigt, dass bisher nur wenige Studien vorliegen, die sich dieser Verbindung widmen – so wurden ältere Menschen nur selten zu ihren Einstellungen und ihrem Wissen über Nachhaltigkeit befragt [12, 20], und es existieren keine Arbeiten dazu, inwieweit nachhaltiges Handeln aus ihrer Sicht ihre Teilhabe beeinflusst. Offen ist auch die Frage, welche Impulse ältere Menschen von der Nachhaltigkeitsstrategie (Agenda 2030) und der Dekade des gesunden Alterns (Zeitrahmen 2021–2030) erhalten. Ein weiteres Desiderat umfasst die Erweiterung der (Alters‑)Forschung im Hinblick auf soziale Ungleichheiten im Klimawandel, z. B. in Ländern des Globalen Südens, die besonders stark von den Folgen betroffen sind. Diese Liste an offenen Fragen und zu erschließenden Feldern verdeutlicht die Brisanz des Themas und die Notwendigkeit, nachhaltiges Alter(n) sowohl konzeptionell als auch empirisch zu be- und ergründen.

## Fazit für die Praxis

Um Handlungsmöglichkeiten im Rahmen des nachhaltigen Alter(n)s zu etablieren, sind mehr Best-Practice-Beispiele vonnöten, die vielfältige Lebensrealitäten adressieren. Impulse dafür geben Klimaschutzbewegungen wie „Omas for Future“ in Deutschland und „KlimaSeniorinnen“ in der Schweiz. Dass diese Formen des Aktivismus hauptsächlich von Frauen getragen werden und Nachhaltigkeit mit der weiblich konnotierten Sorge um nachkommende Generationen verknüpft wird, ist zu reflektieren. Institutionelle Akteur:innen wie die der Sozialen Altenarbeit sind gefragt, damit sie Ältere in der Realisierung eines nachhaltigen Alter(n)s bei Bedarf begleiten und intergenerationale Konflikte zugunsten von gesellschaftlicher Solidarität minimieren.

## Footnotes

1 Wir danken Hanna-Lena Wilmes für die wertvolle Unterstützung bei der Erstellung des Scoping Reviews.

2 Diese zwei Orientierungen werden im Nachhaltigkeitsdiskurs noch weiter ausdifferenziert, z. B. in schwache, ausgewogene und starke Nachhaltigkeit [43].
